# Sodium butyrate treatment and fecal microbiota transplantation provide relief from ulcerative colitis-induced prostate enlargement

**DOI:** 10.3389/fcimb.2022.1037279

**Published:** 2022-10-25

**Authors:** Weimin Dong, Jiefang Zheng, Yiqiao Huang, Huijing Tan, Shengbang Yang, Zhiming Zhang, Xue Liang, Hao Liu, Guohao Zeng, Haoming Xu, Xianhan Jiang, Weide Zhong

**Affiliations:** ^1^ Department of Urology, The Fifth Affiliated Hospital of Guangzhou Medical University, Key Laboratory of Biological Targeting Diagnosis, Therapy and Rehabilitation of Guangdong Higher Education Institutes, Guangzhou, Guangdong, China; ^2^ Department of Urology, Guangdong Key Laboratory of Clinical Molecular Medicine and Diagnostics, Guangzhou First People’s Hospital, School of Medicine, South China University of Technology, Guangzhou, China; ^3^ Department of Gastroenterology and Hepatology, Guangzhou Digestive Disease Center, Guangzhou First People’s Hospital, School of Medicine, South China University of Technology, Guangzhou, Guangdong, China

**Keywords:** sodium butyrate, fecal microbiota transplantation, ulcerative colitis, prostate enlargement, G protein-coupled estrogen receptor (GPER)

## Abstract

The ability to regulate the gut environment has resulted in remarkable great breakthroughs in the treatment of several diseases. Several studies have found that the regulation of the gut environment might provide relief from the symptoms of benign prostatic hyperplasia. However, the correlation between the gut microenvironment and the colon and prostate glands is still unknown. We found that ulcerative colitis (UC) induced an increase in prostate volumes that could be reversed by sodium butyrate (NaB) and fecal microbiota transplantation (FMT). The mechanism by which UC induced changes in the prostate gland was examined *via* RNA-Seq. The results show that the expression level of GPER was significantly lower in the prostate gland of UC mices than in normal mices. The expression of GPER could be increased *via* treatment with NaB or FMT. We found that prostate tissues exhibited higher butryic acid levels after they were treated with NaB or FMT. In experiments conducted *in vitro*, NaB or the fecal filtrate (FF) from healthy mice up-regulated of the expression of GPER, inhibited cell growth, and induced apoptosis in BPH-1 cells. These changes could be alleviated by treatment with the G15 or in GPER-silenced cells.

## Introduction

Benign prostatic hyperplasia (BPH) is associated with dysuria and urinary retention, and is common in elderly people (Woodard et al., 2016). It results in a decreases in the quality of life of patients because of its persistent symptoms and long treatment cycle (Zhang et al., 2022). The regulation of the gut environment is widely used as a treatment with low economic burden in a variety of diseases (Gupta et al., 2020). The consumption of foods such as nuts, fruits, and vegetables which encourage the production of short-chain fatty acids (SCFAs), could help relieve the symptoms of BPH (Russo et al., 2021).

The prostate gland is located near the colon in the body. An inflammation of the colon, known as ulcerative colitis (UC), is similar to BPH. It is difficult to cure and it is easy for the patient to relapse (Ferretti et al., 2022). Alternations in SCFAs and gut microbiota diversity lead to the development of both ulcerative colitis and BPH (Ratajczak et al., 2021; Xu et al., 2022). However, few studies have reported on the relationship between changes in the prostate gland and UC. According to the results of our previous study, butyric acid production levels are correlated with the effect of fecal microbiota transplantation (FMT) during UC treatment (Xu et al., 2021a). Butyric acid, as an SCFA, is the main source of energy for the colon (Pituch et al., 2013). Sodium butyrate (NaB) is a salt of butyric acid (Pituch et al., 2013). We found that NaB and FMT inhibit the progression of UC (Zhao et al., 2020; Xu et al., 2021a; Xu et al., 2021b). In this study, we aim to examine the changes in the prostate gland and explore whether FMT or NaB affects the prostate gland during the progression of UC.

## Materials and methods

### Establishment of a mouse model

All animal experiments were performed in accordance with the protocols used in our previous studies (Xu et al., 2021a). The animal study was reviewed and approved by The institutional review board of Guangzhou Medical University. Sixteen eight-weeks-old male BALB/c mice were obtained from the Animal Center at Guangdong Medical Laboratory. The DSS-induced colitis model symptomatically resembles epithelial damage and inflammatory state in UC. Water containing 2.5% DSS (Sigma, United States) was provided every other week for 5 weeks to induce UC. Mice in the control group were provided water alone. The UC-NaB groups included mice with colitis that were fed with 0.2g/kg NaB for 7 days. The UC-FMT group was gavaged with filtrates of fresh feces obtained from healthy mice from the control group one day at a time, for a total of 7 days. The disease activity index (DAI) was used to grade colitis severity and determine the stool consistency (0=normal, 1=pasty and not sticking to the anus,2=pasty and slightly sticking to the anus, 3=pasty and stuck and to the anus, 4=watery), weight loss percentage (0 = 0-1%, 1 = 1-5%, 2 = 5-10%, 3 = 10-15%, 4 = 15-100%), and extent of rectal bleeding (0= hemoccult (–), 1= hemoccult (±), 2=hemoccult (+), 3=hemoccult (++), 4=obvious blood in stool) (Murthy et al., 1993). Fecal samples (500 mg) were collected from healthy mice in the control group solubilized in PBS, and passed through a 0.2 μm membrane filter (Shute et al., 2021). Prostate volume was calculated by height × length × width × π/6.

### Western blot

Tissue or cell proteins were separated *via* 10% SDS-PAGE and transferred onto 0.22 µm PVDF membranes (Millipore, #ISEQ00010). The membranes were blocked with 5% skimmed milk (BD Difco, #232100) in TBS-Tween 20 and incubated with GPER (Abcam, ab260033) and Anti-β-Actin Antibody (Boster, #BM0627). The results were visualized with the SImmobilonWestern Chemiluminescent HRP substrate (Millipore, #WBKLS0100). β-Actin was used as a control for internal loading.

### Immunohistochemistry

Expression levels and subcellular localization were detected using anti-ZO-1 (Abcam, ab216880), anti-occludin (Abcam, ab168986), and anti-GPER (Abcam, ab260033). Two pathologists who were blinded to the clinical data scored the results of the immunostaining process independently. The scoring scheme was based on the percentage of positive cells (0 = 0%, 1 = 1-10%, 2 = 11-50%, 3 = 50%-80%, 4 = 81%-100%) and the staining intensity (0=no staining, 1=weakly stained, 2=moderately stained, 3=strongly stained). These antibodies were diluted by 1:200 times.

### Gene silencing with shRNAs

Plasmid-targeting GPER shRNA was obtained from the HYY Med company (Guangzhou, China). The framework plasmid was used as a lentiviral vector. BPH-1 cells were infected with GPER-shRNA and its control vector. The shRNA targeting sequences in this study: GPER-shRNA-a:GCAACATCCTGATCCTGGTGGTGAA, 1111; GPER-shRNA-b: CCGACTCCCTCATTGAGGTGTTCAA, 1213; GPER-shRNA-c: CAGGTCAACATGTACAGCAGCGTCT, 1296. Stable cell lines were obtained after the medicinal sieving. The efficacy of these three shRNAs was evaluated by western blot. The sequence with the highest inhibition efficacy for GPER and GPER-shRNA-c, was identified by western blot.

### Sequencing data analysis

Prostate tissue was selected for RNA-Seq. Total RNA was extracted using a Trizol reagent kit (Invitrogen, Carlsbad, CA, USA). RNA quality was assessed using an Agilent 2100 Bioanalyzer (Agilent Technologies, Palo Alto, CA, USA) and checked *via* electrophoresis using RNase-free agarose gel. After total RNA was extracted, eukaryotic mRNA was enriched using Oligo(dT) beads. Then, the enriched mRNA was broken down into short fragments using fragmentation buffer and reverse transcribed into cDNA using the NEBNext Ultra RNA Library Prep Kit for Illumina (NEB #7530, New England Biolabs). The end repair of purified double-stranded cDNA fragments was performed, a base was added, and the fragments were ligated to Illumina sequencing adapters. The ligated sequences were purified using AMPure XP Beads (1.0X) and subjected to PCR amplification and size selection *via* agarose gel electrophoresis. The resulting cDNA library was sequenced using an Illumina Novaseq6000 instrument, which was developed by Gene Denovo Biotechnology Co. (Guangzhou, China). The results suggested that the sample could be used for further analysis. DEGs were mapped to GO terms in the Gene Ontology (http://www.geneontology.org/) and KEGG Pathway (http://www.genome.jp/kegg/) database. The raw sequencing data have been deposited in the Genome Sequence Archive (http://bigd.big.ac.cn/)

### Cell cultures

BPH-1 is a benign prostate epithelial cell line. Cells were purchased from Sigma-Aldrich and authenticated by the HYY Med company (Guangzhou, China). The BPH-1 cell line was maintained in RPMI-1640 medium (HyCloneTM, #SH30027.01). The media were supplemented with 10% fetal bovine serum (Gibco, #10438-026), 2 mM L-glutamine, and 1%antibiotics. Both cell lines were maintained at 37°C and 5% CO2.

### Cell viability

BPH-1 cells were seeded in 96-well plates and treated or not treated with a medium containing 1 mM NaB, 10 μM G15, fecal filtrate, both 1 mM NaB and 10 μM G15,and both fecal filtrate and 10 μM G15. After 24 h, a 10% CCK-8 (Beyotime, C0038) solution was added to each well. A spectrophotometer (Multiskan MK3, Thermo Scientific) was used to detected the absorbance values at 450 nm.

### Cell cycle analysis

BPH-1 cells that were treated or not treated with a medium containing 1 mM NaB, fecal filtrate, both 1 mM NaB and 10 μM G15,and both fecal filtrate and 10 μM G15 for 24 h were collected, washed, and fixed in 70% ethanol at 4°C overnight. A mixture containing PBS, propidium iodide, RNaseA, and 0.3% Triton X-100 was added to the cells and then be incubated at 4°C for half an hour. A flow cytometer (BD FACSVerse, BD Biosciences, USA) and FlowJo software were used to for analysis. Three independent tests were conducted at least three times.

### Apoptosis detection

BPH-1 cells were cultured for 24 h in 5 conditions: medium alone, 1 mM NaB, fecal filtrate, both 1 mM NaB and 10 μM G15,and both fecal filtrate and 10 μM G15 for 24 h. Then, the cells were harvested *via* trypsinization and incubated with Annexin V-APC and 7AAD. After vortexing, the cells were incubated at about 26°C in the dark for a quarter. Then, 485 µL of 1× binding buffer was added to each sample. The results were analyzed with a flow cytometer (BD FACSVerse, BD Biosciences, USA). Three independent tests were conducted at least three times.

### Butyric acid ELISA kit

We collected tissue homogenates from the prostate or colon of mice from the control group, UC group, UC-NaB group, and UC-FMT group. The level of butyric acid (BA) was measured using the BA ELISA Kit, according to the manufacturers’ instructions (Elk biotechnology, ELK8174).

### Statistics

Relevant data are shown in terms of mean ± standard deviation (SD) values. Statistical analysis was performed with independent samples using the t-test, along with SPSS V16 and Graph Pad Prism 8 software. Data results were considered statistically significant if *p < 0.05, **p < 0.01, ***p < 0.001, and ****p<0.0001.

## Results

### NaB or FMT inhibit the progression of UC

**Figure 1:**
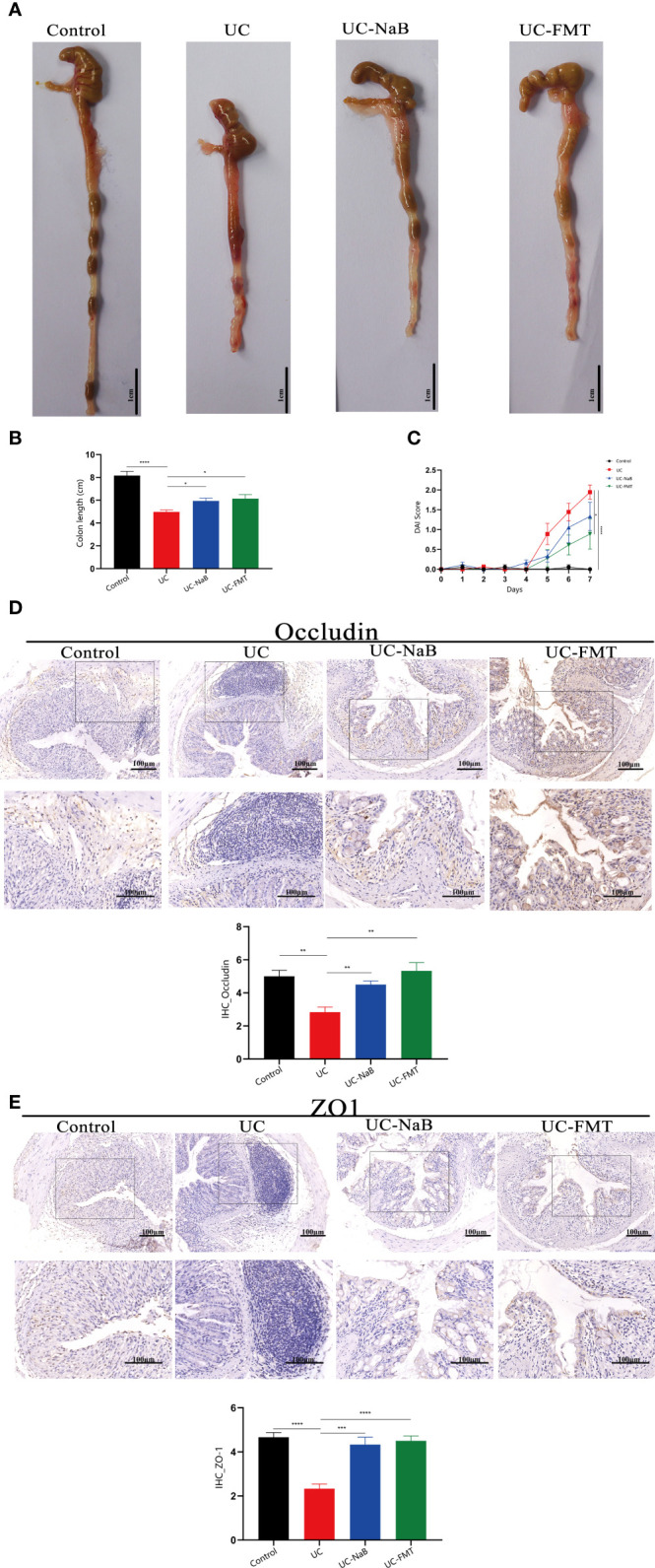
Pathological model of the colon in the mouse. **(A)** Anatomical map of the colorectum. Mouse colon lengths **(B)** and disease activity index (DAI) **(C)** in each group. Occludin **(D)** and ZO1 **(E)** expression in colonic tissues. *p<0.05, **p<0.01, ***p<0.001, ****p<0.0001.

We analyzed and compared the colons of healthy mice and mice with colitis treated or not treated with NaB or FMT. All samples were handled by investigators who were blinded to the data, in accordance with legal and ethical standards. The lengths of colons in the UC group were significantly shorter than in the control, UC-NaB, and UC-FMT groups (**Figures 1A, B**), while the DAI values in the UC group were higher than those in the other groups (**Figure 1C**). Occludin and ZO-1, which are the biomarkers of gut barrier, were negatively correlated at the intestinal inflammation level (Kato et al., 2007). Immunohistochemistry was used to detect the expression levels of occludin (**Figure 1D**) and ZO-1 proteins (**Figure 1E**). As observed for the control group, the expression level of occludin and ZO-1 in the FMT group or NaB group was significantly higher than that in the UC group; this was consistent with our previously reported results.

### NaB or FMT inhibit UC-induced prostate enlargement

**Figure 2:**
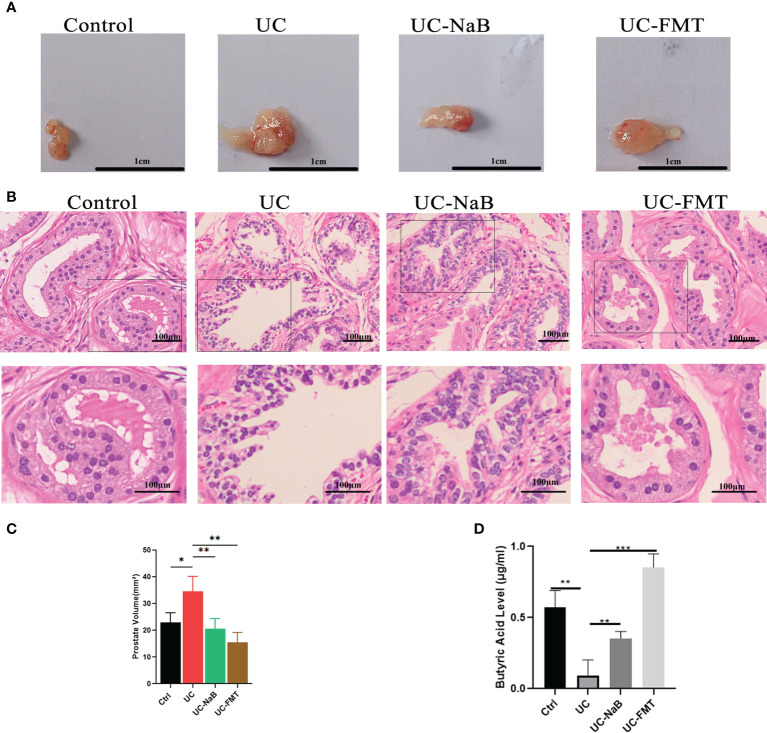
Pathological model of the prostate in the mouse. **(A)** Anatomical map of the prostate. **(B)** Histopathological images of HE staining of the prostate glands. Histopathological images of HE staining **(B)** and volume **(C)** of the prostate glands. **(D)** The levels of butyric acid in prostate gland. *p<0.05, **p<0.01, ***p<0.001.

Prostate tissues were also collected from these animals. The volume of the prostate gland was increased in the UC group when compare to the control group. It was smaller after treatment with NaB or FMT in mice with colitis (**Figure 2A, C**). The results of histological and morphological analyses reveal that the epithelial cell layer and lumen space were increased in the UC group, compared to the control group (**Figure 2B**). These changes were reversed after treatment with NaB or FMT. We detected different levels of butyric acid (BA) levels in the prostate tissues of these mices. The results show that the level of BA was higher in the UC-NaB group and UC-FMT groups than in the UC group (**Figure 2D**).

**Figure 3:**
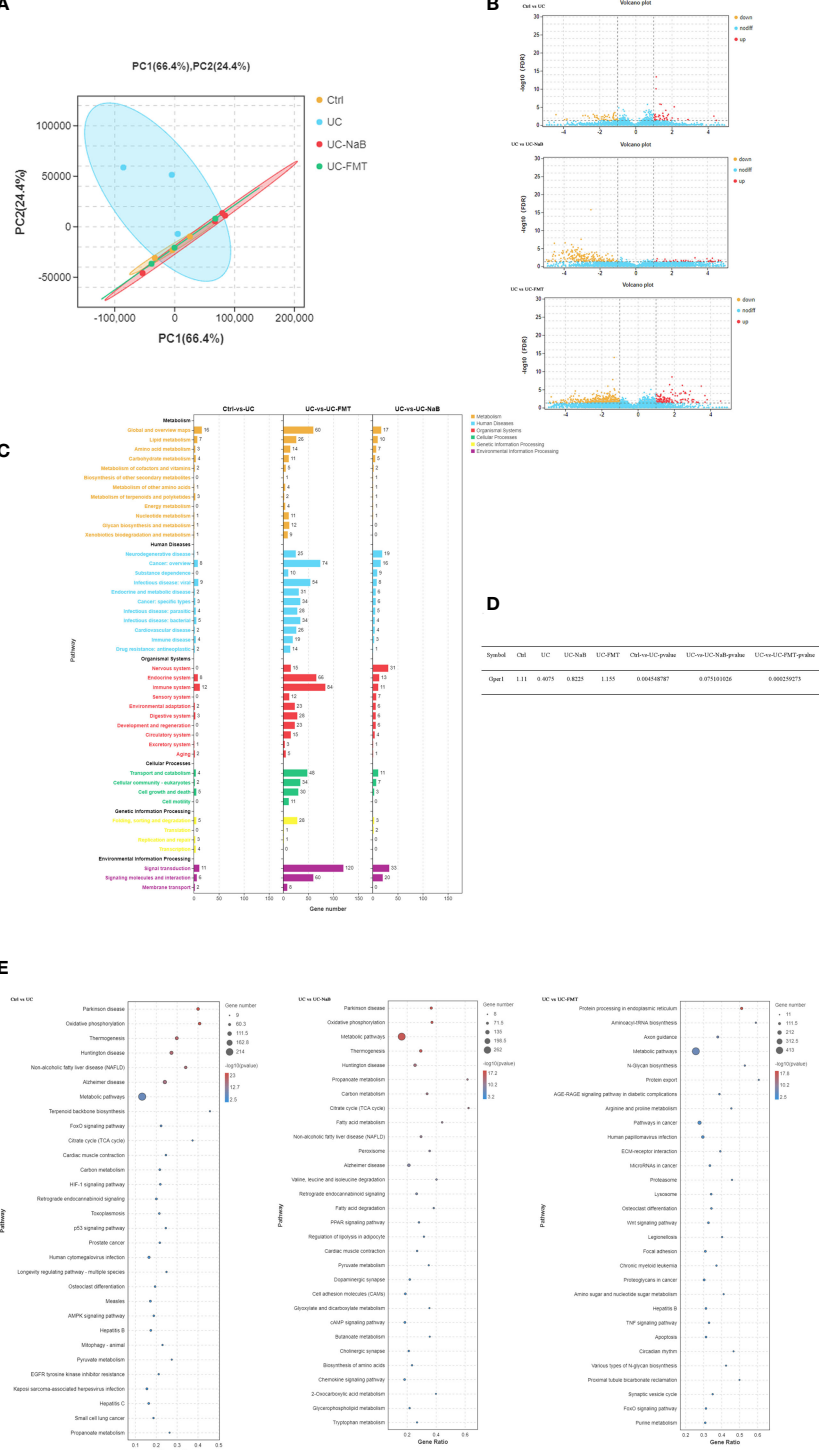
PCA was performed with the R package gmodels (http://www.rproject.org/) in this experiment for each of the sample groups, including their biological replicates. **(A)**. The expression profiles of the identified DEGs **(B)**. Yellow and red points represent significant DEGs with an FDR<0.05 and |log2FC|>1, and blue points indicate non-significance DEGs. **(C)**. The vertical axis represents the number of DEGs per pathway in KEGG annotation. The X-axis shows the gene numbers for each different term. **(D)**. The GPER expression levels in each group were compared. **(E)**. Enriched GO terms identified during the analysis of DEGs.

Sequencing data were analyzed in order to understand the mechanisms underlying the action of NaB and FMT in this model. Principal component analysis (PCA) indicated that there were differences among the control, UC, UC-NaB, and UC-FMT groups (**Figure 3A**). This result suggested that the sample could be used for further analysis. There were 99, 267, and 758 differentially expressed genes (DEGs) between the control and UC group, UC group and UC - NaB group, UC group and UC- FMT group, respectively (**Figure 3B**). Among all the enriched KEGG pathways, after excluding the irrelevant information, some human diseases and metabolism-related pathways are the most significant, including the “cancer pathways”, “liquid metabolism pathway”, and “cell growth and death pathways” (**Figures 3C, E**). We found that G protein-coupled estrogen receptor 1 (GPER/GPR30) was down-regulated in the UC group and responded to all these pathways (**Figure 3D**). GPER is a member of the GPCR family of proteins. We have demonstrated that the activation of GPER inhibits BPH-1 cells proliferation (Dong et al., 2019).

### GPER associated with UC-induced prostate enlargement

**Figure 4:**
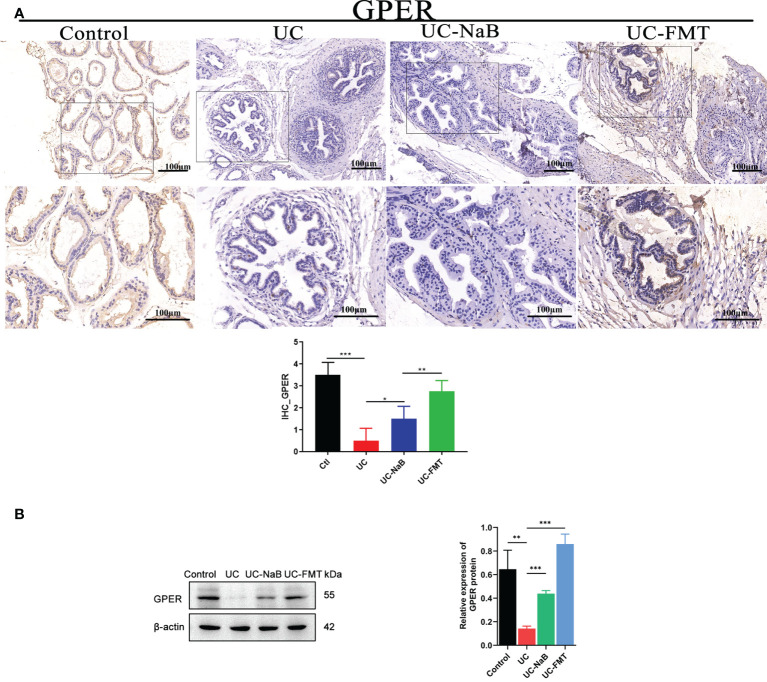
GPER expression in the prostate tissue. Immunohistochemistry **(A)** and western blot **(B)** analysis of proteins in the prostate tissue under different conditions. *p<0.05, **p<0.01, ***p<0.001.

We examined the levels of GPER in prostate tissues *via* immunohistochemistry (**Figure 4A**) and western blot (**Figure 4B**) analyses. The level of GPER in the UC-FMT, UC-NaB, or control groups was significantly higher than that in the UC group, which is consistent with the results of RNA sequencing.

**Figure 5:**
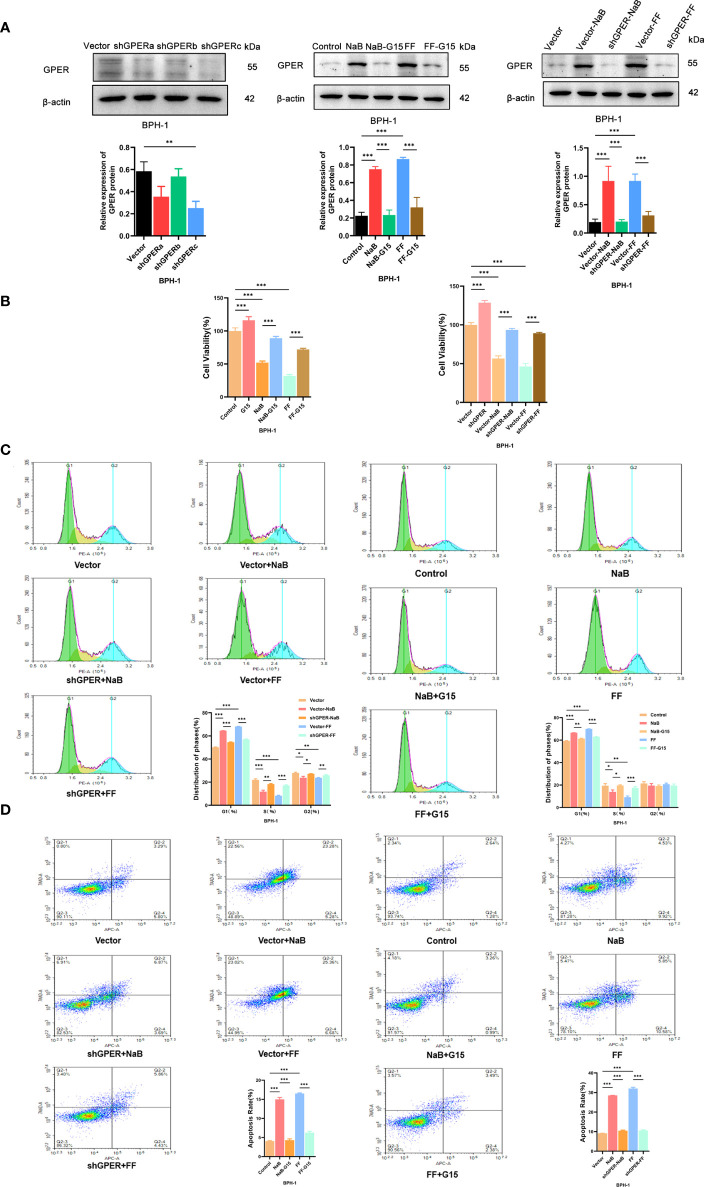
**(A)**. Western blot of proteins in BPH-1 cells. **(B)**. Cell viability assays were conducted with BPH-1 cells under different conditions. **(C)**. Cell cycle analysis results for the control, NaB, NaB+G15, FF, FF+G15, Vector, Vector+NaB, shGPER+NaB, Vector+FF, and shGPER+FF groups. **(D)**. Cell apoptosis assay results of the Control, NaB, NaB+G15, FF, FF+G15, Vector, Vector+NaB, shGPER+NaB, Vector+FF, and shGPER+FF groups. *p<0.05, **p<0.01, ***p<0.001.

The sh-GPER cell line was established and verified by western blot analyses (**Figure 5A**). The GPER expression levels observed *in vitro* are higher in BPH-1 cells treated with NaB or filtrates of fresh feces from healthy mice (**Figure 5A**), compared with the control group. Such upregulation of GPER appears to be weaker in GPER-silencing group or cells treated with GPER antagonist G15. Cell growth is affected by cell proliferation, cell cycle, and apoptosis. The results of the CCK-8 assay (**Figure 5B**) showed that NaB or FF inhibited the growth of BPH-1 cells. The cell proliferation assay results showed that these stimuli increased the percentage of G1 phase cells (**Figure 5C**). We also found that NaB and FF induce apoptosis in the BPH-1 cells (**Figure 5D**). However, the extent of induction of apoptosis, inhibition of cell proliferation, and prolongation of the G1 phase appeared to be reduced in GPER-silenced cells or cells treated with G15 (**Figures 5B–D**). Therefore, we believe that GPER plays an important role in the enhancement of cell growth that was inhibited by NaB or FMT in BPH-1 cells.

## Conclusion

Our study provides a better understanding of the intestinal microenvironment affects the process of BPH development. UC-induced prostate enlargement which could be inhibited by NaB and FMT, and might be correlate to cancer and metabolic pathway. NaB and FMT might inhibit prostate growth by resulting in the expression of GPER.

## Discussion

The change in the diversity of gut bacteria in patients has attracted considerable attention from researchers. Not only do gut bacteria act as novel biomarkers, but they also act as novel disease-modifying targets (Durack and Lynch, 2019; Manor et al., 2020). A recent study reported on the change in gut bacteria in BPH patients (Li et al., 2022). The existence of the gut-genitourinary axis was also proposed in this study (Li et al., 2022). However, the mechanism by how gut affect prostatic diseases is still relatively unknown.

Both BPH and UC are diseases that occur repeatedly and necessitate long-term treatment. FMT and postbiotics are promising potential therapies, especially for relapsing diseases, because of their relatively fewer side effects and low toxicity levels (Yoshimatsu et al., 2015; Salminen et al., 2021; Mendelsohn et al., 2022). Butyrate has been considered as a postbiotic (Panebianco et al., 2022). The colon is the main site at which butyric acid absorption and gut microbiological colonization occur. We analyzed 12 UC patient samples in our previous study and found that the FMT efficacy was associated with the level of butyric acid-producing bacteria (Xu et al., 2021a). Here, we not only verified that supplementary butyrate treatment or FMT had a positive effect on ulcerative colitis again but also found that NaB and FMT suppressed UC-induced prostate enlargement. The period at which BPH and ulcerative colitis can be predicted is different. This might be attributable to the fact that it is difficult to detect a slightly enlarged prostate at an early stage. Subsequently, butyrate enters the circulation and metabolizes substrates (Zhang et al., 2021). Therefore, FMT is more beneficial because it results in a higher level of butyric acid in the systemic circulation; this needs to be verified in future studies. We believe that NaB treatment and FMT could lower the risk of BPH. Therefore, FMT might represent a potential new therapy for the treatment of BPH.

We performed RNA sequencing to further examine how FMT and NaB affected UC-related prostate changes in the prostate. We found that UC-related changes in the prostate were correlated with cancer and the metabolic pathway. Though SCFAs activate multiple GPCRs, the mechanism by which butyrate affects GPER is still unknown (Park et al., 2019). The direct effects of bacteria in the gut and changes in the BA concentration may results in a change in the prostate. We verified that NaB and filtrates of fresh rat dejecta up-regulated the expression of GPER and promoted the process of cellular apoptosis, which could be suppressed in GPER-silenced cells. This indicated that GPER was one of the most significant factors affecting this process. The detailed mechanisms of action need to be further elucidated at the molecular level, to uncover the relationship between NaB and FF. This study provides a better understanding of the mechanism of action of GPER in the gut-genitourinary axis. Butyrate and FMT may be a protential therapy for UC-induced prostate enlargement. The regulation of GPER levels might help to improve the therapeutic effects of FMT and NaB with BPH.

## Data availability statement

The data presented in the study are deposited in the Genome Sequence Archive (http://bigd.big.ac.cn/), accession number: CRA008379.

## Ethics statement

The animal study was reviewed and approved by the institutional review board of Guangzhou Medical University.

## Author contributions

WD, XJ, and HX designed the study. WD, JZ, HX, HT, and SY established the animal model and collected the samples. JZ, YH, ZZ, XL, HL, and GZ carried out the experiments and analyzed the data. WD, XJ, HX, and WZ drafted and revised the article. All authors have read and approved the final manuscript.

## Funding

This work was supported by grants from National Natural Science Fund of China (82072808), Natural Science Fund of Guangdong Province (2019A1515010222), Guangzhou Core Medical Disciplines Project (2021-2023), Guangzhou Municipal Science and Technology Bureau Municipal finance - Supporting Institution Jointly funded project Funds (202102010137), and Guangzhou Municipal Science and Technology Project (202102020848).

## Conflict of interest

The authors declare that the research was conducted in the absence of any commercial or financial relationships that could be construed as a potential conflict of interest.

## Publisher’s note

All claims expressed in this article are solely those of the authors and do not necessarily represent those of their affiliated organizations, or those of the publisher, the editors and the reviewers. Any product that may be evaluated in this article, or claim that may be made by its manufacturer, is not guaranteed or endorsed by the publisher.
